# A Novel RNA Editing Sensor Tool and a Specific Agonist Determine Neuronal Protein Expression of RNA-Edited Glycine Receptors and Identify a Genomic APOBEC1 Dimorphism as a New Genetic Risk Factor of Epilepsy

**DOI:** 10.3389/fnmol.2017.00439

**Published:** 2018-01-11

**Authors:** Svenja Kankowski, Benjamin Förstera, Aline Winkelmann, Pina Knauff, Erich E. Wanker, Xintian A. You, Marcus Semtner, Florian Hetsch, Jochen C. Meier

**Affiliations:** ^1^Division Cell Physiology, Zoological Institute, Technische Universität Braunschweig, Braunschweig, Germany; ^2^Institute for Stroke and Dementia Research, Klinikum der Universität München, Ludwig Maximilians University of Munich, Munich, Germany; ^3^Neuroproteomics, Max Delbrueck Center for Molecular Medicine, Berlin, Germany; ^4^Institute of Cell Biology and Neurobiology, Charité Universitätsmedizin Berlin, Berlin, Germany; ^5^Bioinformatics in Medicine, Zuse Institute Berlin, Berlin, Germany; ^6^Cellular Neurosciences, Max Delbrueck Center for Molecular Medicine, Berlin, Germany

**Keywords:** glycine receptors, epilepsy, temporal lobe, RNA editing, hippocampus, ligands

## Abstract

C-to-U RNA editing of glycine receptors (GlyR) can play an important role in disease progression of temporal lobe epilepsy (TLE) as it may contribute in a neuron type-specific way to neuropsychiatric symptoms of the disease. It is therefore necessary to develop tools that allow identification of neuron types that express RNA-edited GlyR protein. In this study, we identify NH_4_ as agonist of C-to-U RNA edited GlyRs. Furthermore, we generated a new molecular C-to-U RNA editing sensor tool that detects Apobec-1- dependent RNA editing in HEPG2 cells and rat primary hippocampal neurons. Using this sensor combined with NH_4_ application, we were able to identify C-to-U RNA editing-competent neurons and expression of C-to-U RNA-edited GlyR protein in neurons. Bioinformatic analysis of 1,000 Genome Project Phase 3 allele frequencies coding for human Apobec-1 80M and 80I variants showed differences between populations, and the results revealed a preference of the 80I variant to generate RNA-edited GlyR protein. Finally, we established a new PCR-based restriction fragment length polymorphism (RFLP) approach to profile mRNA expression with regard to the genetic *APOBEC1* dimorphism of patients with intractable temporal lobe epilepsy (iTLE) and found that the patients fall into two groups. Patients with expression of the Apobec-1 80I variant mostly suffered from simple or complex partial seizures, whereas patients with 80M expression exhibited secondarily generalized seizure activity. Thus, our method allows the characterization of Apobec-1 80M and 80l variants in the brain and provides a new way to epidemiologically and semiologically classify iTLE according to the two different *APOBEC1* alleles. Together, these results demonstrate Apobec-1-dependent expression of RNA-edited GlyR protein in neurons and identify the *APOBEC1* 80I/M-coding alleles as new genetic risk factors for iTLE patients.

## Introduction

RNA editing plays an important role in the diversification of gene products. There are two different types of enzymatic deamination which lead either to the conversion of adenosine to inosine (A-to-I RNA editing) or of cytidine to uridine (C-to-U RNA editing). While the role of A-to-I RNA editing in health and disease is being investigated by many laboratories, research on C-to-U RNA editing is still at a rudimentary level (Meier et al., 2016).

In this study, we focused on C-to-U RNA editing and the involvement of the apolipoprotein B (ApoB) mRNA editing enzyme catalytic polypeptide-like Apobec-1. Successful cloning of *APOBEC-1*, the first identified member of the *APOBEC* gene family of cytidine deaminases (Navaratnam et al., 1993; Teng et al., 1993), was a milestone in research on C-to-U RNA editing. The role of *APOBEC1* and auxiliary proteins in pre-mRNA editing of ApoB in liver and small intestine was elucidated in great detail (Backus and Smith, 1992; Schock et al., 1996; Dance et al., 2002; Smith, 2007). However, C-to-U RNA editing may also play a critical role in the central nervous system. In fact, C-to-U RNA editing of gene transcripts coding for the neurotransmitter receptor for glycine (GlyR) was discovered over 10 years ago (Meier et al., 2005). In the case of GlyR, C-to-U RNA editing leads to amino acid substitution of leucine for proline and receptor gain-of-function (Meier et al., 2005; Legendre et al., 2009).

Using bulk material of resected hippocampi, GlyR C-to-U RNA editing was shown to be increased in patients with severe intractable temporal lobe epilepsy (iTLE) (Eichler et al., 2008). Increased C-to-U RNA editing leads to neuronal gain-of-function through presynaptic activity of RNA-edited GlyRs, resulting in facilitation of neurotransmitter release (Bischofberger et al., 2006; Meier et al., 2014). This ultimately and persistently tilts the balance between excitation and inhibition in a bidirectional way (Eichler and Meier, 2008). Depending on the neuron type that is afflicted with increased GlyR C-to-U RNA editing, cognitive dysfunction or persistence of contextual fear memory can thus arise through enhanced function of glutamatergic or parvalbumin type GABAergic neurons, respectively, which has been shown in a corresponding animal model of the disease (Winkelmann et al., 2014; Çaliskan et al., 2016). Thus, methods for neuron type-specific detection of RNA-edited GlyR protein are ultimately required to elucidate the role of this gain-of-function neurotransmitter receptor in iTLE.

We present here novel molecular and chemical tools for detection of C-to-U RNA editing at the single cell level. Furthermore, the results identify an agonist that allows discrimination between RNA-edited and non-RNA-edited GlyR proteins in hippocampal neurons. The combination of these novel molecular and chemical tools enable a proof-of-principle demonstration of the role of Apobec-1 in C-to-U RNA editing of GlyR and expression of the RNA-edited GlyR protein. Moreover, bioinformatic analysis revealed worldwide differences in the allelic distributions of two different *APOBEC1* 80I- or 80M-coding alleles. Using a new PCR-based restriction fragment length polymorphism (RFLP) approach we furthermore provide a retrospective assessment of the iTLE patients analyzed in 2008 (Eichler et al., 2008) with regard to expression of Apobec-1 80I and 80M variants. The results showed that iTLE patients with 80I expression mostly experienced simple or complex partial seizures, whereas patients with 80M expression exhibited secondarily generalized seizure activity. This establishes the basis for a new mapping of epidemiology and semiology of iTLE with regard to *APOBEC1* 80M/I alleles and opens up new ways to functionally characterize the Apobec-1 80M and 80I variants in the brain and in iTLE patients. Together, these results advance our understanding of C-to-U RNA editing at the single cell level and establish expression of the Apobec-1 80I and 80M variants as new genetic risk factors of iTLE.

## Methods

### Molecular constructs

**Figure 1:**
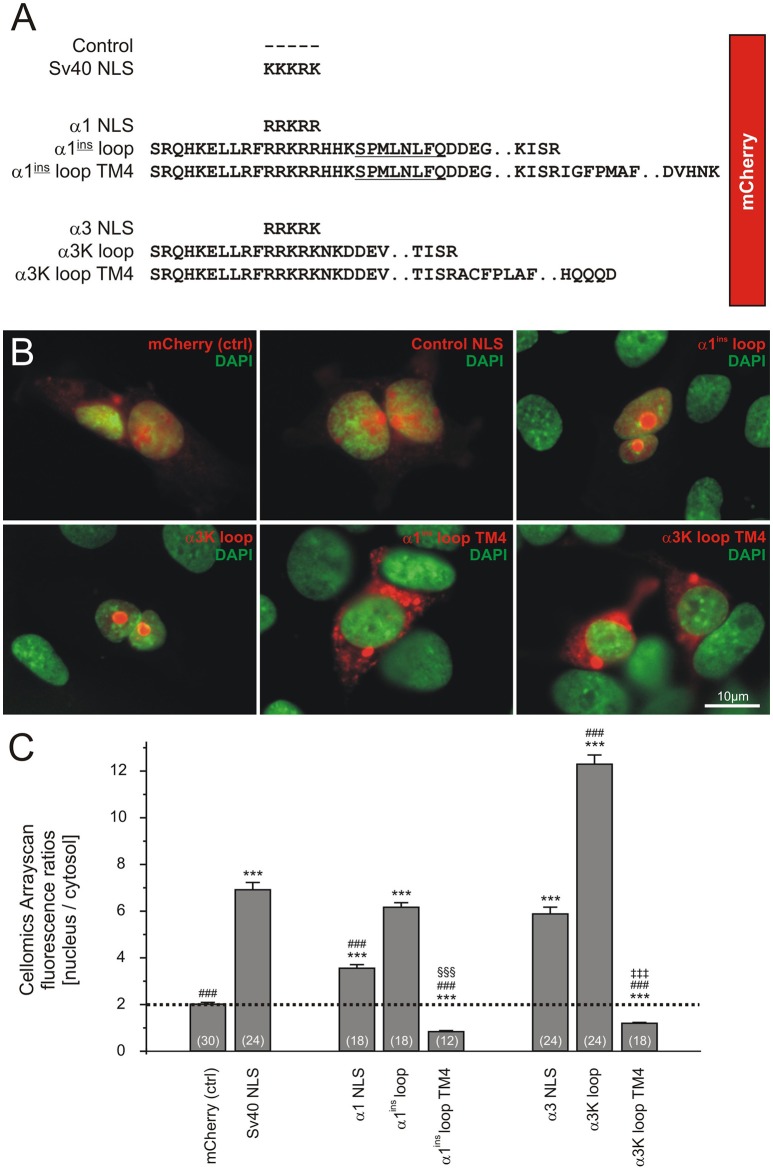
The GlyR transmembrane domain 4 (TM4) determines cytosolic localization of the GlyR TM3-4 loop in HEK293T cells. **(A)** Design of molecular constructs. Upstream of mCherry, different sequences were cloned including SV40 NLS (KKKRK), GlyR α1 loop NLS (RRKRR), and GlyR α1^ins^ TM3-4 without or with the TM4. The respective constructs were also established for the GlyR α3 subunit. **(B)** Representative images showing fluorescence signals of mCherry constructs (red) and DAPI (green). **(C)** Quantification of the fluorescence ratios (nucleus/cytosol) of mCherry signals using Cellomics plate reader software in live cells stained with Hoechst 33342. Note that the most pronounced difference between nucleus/cytosol ratios of GlyR TM3-4 constructs with or without the TM4 domain was observed for the GlyR α3 subunit. Significance levels are given for conditions compared to control (^*^), compared to SV40-NLS (#), compared to α1^ins^ loop (§) and compared to α3K loop (‡). In all cases, three symbols mark statistical significance (*P* < 0.001), as assessed using Mann-Whitney test. At least 1,000 cells per condition and cell culture plate well were analyzed. The number of wells analyzed in 3 independent experiments is indicated in brackets (see Table 1).

SV40 nucleus localization sequence-containing clones and expression constructs containing the large cytosolic loops between transmembrane domains TM3 and TM4 of GlyR α1^ins^ and α3K were amplified with PCR and cloned in frame with the mCherry coding sequence of an in-house made vector derived from EGFP-N1 (Clontech), using EcoRI and AgeI restriction enzymes (Figure 1A). Corresponding cDNA clones (Legendre et al., 2009) served as PCR templates. For construction of the C-to-U RNA editing sensor, the Apobec1-consensus motive was inserted upstream of the GlyR α3K TM4 domain using fusion-PCR and BclI-restriction digest; BclI cleaves within the Apobec1-consensus sequence. The resulting editing sensor construct thus consists of the large cytosolic loop between TM3 and TM4 of GlyR α3K followed by the Apobec1-consensus sequence and the TM4. It reports C-to-U RNA editing according to the nuclear translocation of mCherry, which changes due to C-to-U RNA editing-dependent premature STOP and lack of TM4. Further constructs were generated in which mCherry was replaced with EGFP.

Apobec-1 complementation factor (ACF) and Apobec1-80I or -80M vectors were purchased from Origene (Rockville, MD 20850, USA). The respective open reading frames (ORFs) were subcloned into our in-house-made *Thosea Asigna* virus 2A-peptide coding vectors (Winkelmann et al., 2015) and finally also cloned upstream of the editing sensor-coding domain using standard cloning techniques and the 2A-peptide as molecular interface.

The EGFP-2A-GlyR α2-192P and -192L constructs were obtained by cloning an EGFP-2A fragment upstream of the GlyR ORF.

All final constructs were verified by DNA sequencing.

### RT-PCR analysis of C-to-U RNA editing enzymes in primary rat hippocampal neurons

Total RNA was isolated from primary hippocampal neurons from E18 rat embryos (DIV8) and reverse transcribed into cDNA as described earlier (Raltschev et al., 2016). Oligos were used to amplify regions of AID (5′-GTCCGCTGGGCTAAGGGTC-3′ and 5′-GCACAGTCGTAGCAGGGGC-3′), APOBEC-1 (5′-CCCTGTAGCTGTTGATCCCACTC-3′ and 5′-CAGAGTTACATGGGGGTATCGGC-3′), ABOBEC-2 (5′-CTGATCGATCTGCCGCCCTTC-3′ and 5′-CAGGTTCTTGGTCTTGCTGAGGG-3′), APOBEC-3 (5′-GCCATCGCAGACCCTATTCACCG-3′ and 5′-CTTGCTGCAGGGGCTCCAGG-3′), APOBEC-4 (5′-GGCAGGGGAGGTGAGTCTGG-3′ and 5′-GTTAGCCTCGTCACAAGGGGAG-3′), ACF (5′-TTCTGTCAGAGGGGCTGCG-3′ and 5′-AGCTTTGGGGGTGTGAAAGG-3′) and GAPDH (5′-CAGTATGACTCTACCCACGG-3′ and 5′-CTCAGTGTAGCCCAGGATG-3′). PCR products were analyzed using electrophoresis with 1.2% agarose gels. Ethidium bromide was used to stain DNA bands.

### PCR-RFLP analysis of human TLE samples

Resected hippocampal tissue of human iTLE patients (Eichler et al., 2008) was analyzed with regard to *APOBEC1* gene dimorphism coding for 80M or 80I Apobec-1 protein variants. For this purpose, we developed a new PCR-based RFLP approach. Total RNA was isolated and reverse transcribed into cDNA as described earlier (Raltschev et al., 2016). Pre-amplification of Apobec-1 was performed using oligonucleotides 5′-CTTCAACCGGTGACCCCACTC-3′ and 5′-TGCGTACAACATCATCCACAGAGG-3′. Then, 3.5 μl of the pre-PCR were investigated in another PCR using oligonucleotides 5′-GAGTTTGACGTCTTCTATGACCC-3′ and 5′-GTTGACAAAATTCCTCCAGCAG-3′ to amplify a region spanning the 80M/I-coding position. This nested PCR amplification step yielded sufficient amount of DNA that was purified with Monarch® DNA Gel Extraction Kit (catalog no. L1020L, New England Biolabs GmbH) and digested using NlaIII restriction enzyme. NlaIII cuts at the 80M-coding position (CATG), and restriction fragments were separated using electrophoresis with 5% agarose gels to identify the genotype of the iTLE patients. For control purpose, Apobec-1 80I- or 80M-coding vectors for transfection were processed in parallel. Ethidium bromide was used to stain DNA bands.

### Cell culture and transfection

HEK293T cells and HEPG2 cells (generous gift by Dr. Markus Kaiser, Department of Chemical Biology, University of Duisburg-Essen, Germany) were cultured in T75 culture flasks containing 10 ml of DMEM (catalog no. 41965-062, Gibco) supplemented with 4.5 g/liter glucose, 10% FCS (catalog no. 1050064, Life Technologies), and 1% penicillin/streptomycin (catalog no. 15140122, Life Technologies) at 37°C and 5% CO_2_ in a humidified incubator. Cell passaging was performed every 3–4 days at an average confluence of 80–90%.

For high-throughput image cytometry (Figure 1), HEK293T cells were transfected at 70% confluency using a standard Ca^2+^/phosphate protocol with 2 μg per 35 mm cell culture dish. Transfected cells were plated 1 day after transfection in black 96-well flat bottom Falcon microplates (BD, Franklin Lakes, NJ, USA) at a density of 10,000 cells per well.

For analysis of subcellular C-to-U RNA editing sensor distribution (**Figure 3**), HEPG2 cells were seeded onto uncoated glass coverslips (diameter 13 mm) 1 day before transfection. Initial cell density was 50,000/cm^2^. Transfection of 2.5 μg DNA was performed at 60–70% confluence with Viafect™ Transfection Reagent (catalog no. E4981, Promega) according to the manufacturer's instructions.

Regarding HEK293T cells used for electrophysiology, 200,000 cells were seeded 2–3 days before transfection onto 35-mm culture dishes containing 1.5 ml of DMEM/FCS/penicillin/streptomycin to reach 90–100% confluence for transfection with FuGENE® HD transfection reagent (catalog no. 04 709 705 001, Roche Applied Science) according to the manufacturer's instructions. For HEK293T cells, 1 μg of DNA was used per transfection. For electrophysiological recordings, transfected HEK293T cells were seeded onto glass coverslips (diameter 13 mm) coated for 20–30 min with 0.1% polyornithine (poly-dl-ornithine hydrobromide, catalog no. P8638-100MG, Sigma). Cells were allowed to adhere for at least 2 h before electrophysiological recordings were carried out.

Primary hippocampal neuron cultures from E18 Wistar rats were prepared as previously described (Winkelmann et al., 2014) according to the permit given by the Animal Care Committee of the Technical University Braunschweig (Zentrale Einrichtung für Tierhaltung der TU Braunschweig, §4 10.15.M TSB TU BS) and maintained in B27- and 1% FCS-supplemented Neurobasal medium (Brewer and Cotman, 1989). The initial cell density was 68,000/cm^2^. Transfection and protein expression were carried out as described (Winkelmann et al., 2014) on DIV6-9 using Effectene transfection reagent (Qiagen, Hilden, Germany). For transfection, coverslips were transferred to wells containing transfection medium (Neurobasal supplemented with 0.25 mM L-glutamine) and incubated with complexes formed with 5 μl of Effectene transfection reagent (Qiagen, Hilden, Germany) and 300 ng of DNA. If co-transfection was performed, editing sensor and APOBEC1-80I or -80M were mixed using 150 ng, each. After transfection, coverslips were transferred to dishes containing expression medium based on glycine-free MEM, supplemented with B27, 1% FCS, vitamin B12 and 0.25 mM L-glutamine, 20 mM D-glucose, 25 μM β-mercaptoethanol, 230 μM Na-pyruvate and 1% penicillin/streptomycin.

### Immunocytochemistry and quantitative image analysis

High-throughput image cytometry of transfected HEK293T cells (Figure 1) was performed using Thermo Scientific Cellomics Arrayscan VTI HCS Reader and the BioApplication Compartemental Analysis protocol of the corresponding software (Thermo Fisher Scientific, Waltham, MA, USA). For this purpose, cell nuclei were stained and identified using 1:2,500-diluted Hoechst 33342 (Invitrogen, Life Technologies, Carlsbad, CA, USA).

For analysis of subcellular C-to-U RNA editing sensor distribution (**Figures 3**, **4**), transfected cells (HEPG2 or primary hippocampal neurons) were fixed 1 day after transfection using a mixture of ice-cold 4% paraformaldehyde and 4% sucrose. HEPG2 cells were mounted using DAPI-containing Vectashield (Vector Laboratories, Burlingame, CA, USA). Primary neurons were permeabilized using 0.12% Triton X100 and further processed for immunocytochemistry with guinea pig antibody against MAP2 (catalog no. 188004, Synaptic Systems) and donkey anti-guinea pig CY5 (catalog no. 706-175-148, Jackson ImmunoResearch) to identify neurons, before mounting in Vectashield with DAPI.

Using Metamorph software (Molecular Devices, Sunnyvale CA), line scans were applied so that the lines covered cytosol and nucleus of transfected cells (**Figures 3**, **4**). Fluorescence intensity values along the scanned lines were acquired using Excel software (Microsoft, Redmond WA, USA). Subsequently, the pixel intensity values were divided into cytosolic and nuclear parts of the cell according to the DAPI signal. Finally, the ratio between mean nuclear and cytosolic pixel intensities per cell was calculated.

### Electrophysiology

Transfected neurons were recorded between DIV7 and 15. A ListMedical amplifier, an ITC-18 interface, and Patchmaster software (HEKA, Lamprecht, Germany) were used for patch clamp recordings and data acquisition. Patch pipettes, made from borosilicate glass (Science Products, Hofheim, Germany), had resistances of 3–7 MΩ when filled with the intracellular solution containing (in mM): CsCl (130), NaCl (5), CaCl_2_ (0.5), MgCl_2_ (1), EGTA (5), and HEPES (30), pH 7.2 (CsOH). The standard extracellular solution contained (in mM): NaCl (140), KCl (5), MgCl_2_ (1), CaCl_2_ (2), HEPES-NaOH (10) and glucose (10), pH 7.4 (NaOH). Cells were clamped at a potential of −50 mV. Series resistances (R_s_), monitored by −5 mV voltage pulses (50 ms) applied every 5 s, were between 10 and 40 MΩ. In the figures, the responses to the voltage pulses are not shown, resulting in a discontinuous appearance of current traces. However, for better legibility of the figures we filled these gaps in displayed recording traces. Experiments with more than 25% change in R_s_ throughout the recording were discarded. Data were acquired at a sampling rate of 10 kHz after filtering at 2.8 kHz. Transfected cells were identified according to EGFP or mCherry fluorescence. If not stated otherwise, voltage clamp data were recorded in standard extracellular solution in the presence of tetrodotoxin (TTX, 0.3 μM; Sigma), D-aminophosphonovaleric acid (APV, 50 μM; Cayman Chemical), 6,7-dinitroquinoxaline-2,3-dione (DNQX, 10 μM, Cayman Chemical), and bicuculline methiodide (20 μM; Sigma). This drug mixture called ABDT was used in most experiments and supplemented with ammonium chloride (0.5–50 mM), strychnine (1 or 10 μM) and glycine (100 μM), where indicated.

Perfusion of the extracellular solutions was gravity-driven. A perfusion pencil with a 250-μm tip placed at a distance of 100–200 μm from the recorded cell was used to obtain relatively short wash-in/wash-out times (<1 s). Analysis of patch clamp data was performed with in-house software written in IGOR 6.37 (WaveMetrics, Lake Oswego, USA) by M. Semtner. Live imaging of transfected neurons was done before establishing contact with the patch pipette using a Zeiss Axioscope 10 (Carl Zeiss, Jena, Germany) equipped with a 40x objective, a custom made mCherry and EGFP filter set (Chroma Technology GmbH), an Uniblitz electronic shutter and a Spot Pursuit 2M pixel monochrome CCD camera. Exposure times ranged between 1 and 3 s, and images were acquired with Metamorph 7.1 (Molecular Devices).

### Statistics

Normal distribution of data was assessed using Shapiro-Wilk test. If normal distribution was given, data was subjected to one-way ANOVA with *post-hoc* Tukey-test. If normal distribution was not given, data was subjected to Mann-Whitney test. If not stated otherwise, all data are given as mean ± SEM.). Asterisks used to indicate statistical significance were defined as follows: “n.s.” (not significant) = *P* > 0.05; one symbol = *P* < 0.05; two symbols = *P* < 0.01; three symbols = *P* < 0.001.

## Results

Recent studies identified the intracellular large cytosolic loop between transmembrane domains 3 and 4 (TM3-4 loop) as a highly efficient nuclear targeting domain (Melzer et al., 2010; Förstera et al., 2014). Here, we revisited and corroborated these findings and furthermore show that addition of the transmembrane domain 4 (TM4) efficiently prevents nuclear import of GlyR α1 and α3 TM3-4 loops and hence is a dominantly acting cytosol-retaining domain. Figure 1A shows the molecular design of the constructs used here. We used TM3-4 loops of GlyR α1^ins^ and α3K splice variants as described (Förstera et al., 2014). These domains were cloned downstream of mCherry. For control purpose, mCherry alone or equipped with SV40 NLS (KKKRK) were used. Furthermore, we included the TM4 in the TM3-4 loop constructs of GlyR α1^ins^ and α3K. Figure 1B shows representative images of transfected HEK293T cells. Note that the GlyR α1^ins^ and α3K TM3-4 loops (red) efficiently targeted the nucleus (green), while the TM4 domain efficiently overrode the nuclear targeting activity of the TM3-4 loops and led to almost exclusive cytosolic localization of the mCherry signal (Figure 1C). Quantification of the ratio between nuclear and cytosolic fluorescence intensities and statistical analysis demonstrated the dominant effect of the TM4 over TM3-4 loops (Figure 1, Table 1).

**Table 1:** Assessment of nuclear targeting capacity of large cytosolic GlyR loop domains.

	**Control**	**SV40-NLS**	**α1-NLS**	**α1^ins^ loop**	**α1^ins^ loop TM4**	**α3-NLS**	**α3K loop**	**α3K loop TM4**
N	30	24	18	18	12	24	24	18
Mean	2.11	6.85	3.59	6.74	0.86	5.99	12.05	1.22
SD	0.29	1.16	0.59	1.07	0.10	1.02	1.54	0.08
SEM	0.05	0.24	0.14	0.25	0.03	0.21	0.31	0.02

*The table shows values corresponding to ratios of nuclear to cytosolic fluorescence intensities in transfected HEK293T cells. N, number of wells; SD, standard deviation; SEM, standard error of the mean*.

**Figure 2:**
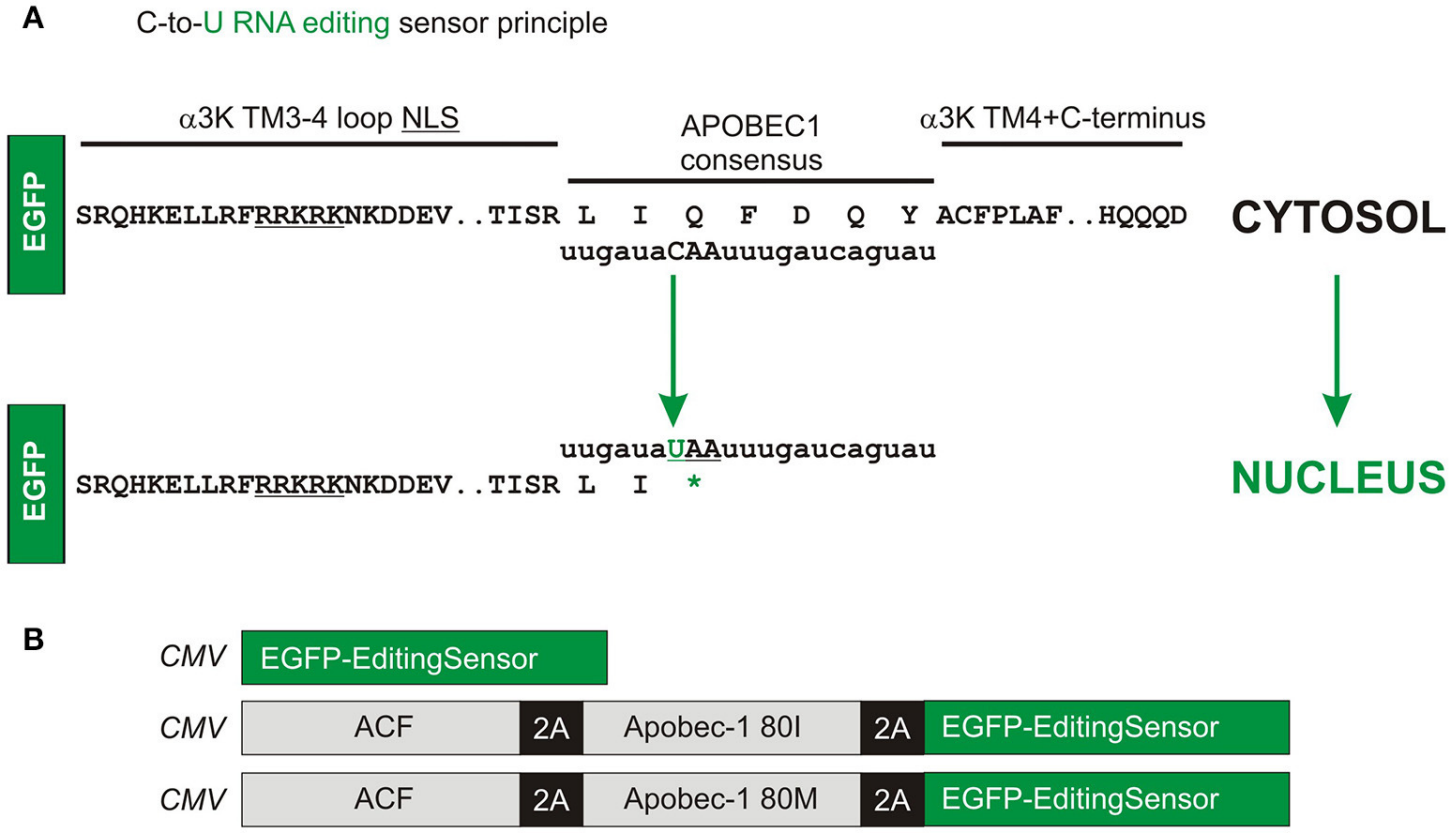
Molecular design of the C-to-U RNA editing sensor. **(A)** The human Apobec-1 consensus sequence was inserted downstream of EGFP-coding sequence between the C-terminal end of the TM3-4 loop of GlyR α3K and the GlyR α3 TM4 domain. Editing of the CAA codon to the STOP codon UAA should result in nuclear targeting of the reporter fluorescence protein (here EGFP). **(B)** Using the *Thosea asigna* virus 2A self-processing peptides, additional constructs with human ACF and Apobec-1 (80I or 80M variants) were generated in order to test the efficacy of the editing sensor in response to equimolar multicistronic expression of the corresponding enzyme complex subunits.

These clear-cut results prompted us to develop a C-to-U RNA editing sensor (Figure 2). As the GlyR α3K TM3-4 with and without TM4 provided most accentuated results regarding nuclear vs. cytosolic targeting, the human Apobec-1 consensus sequence derived from apolipoprotein B was inserted between the C-terminal end of the TM3-4 loop of GlyR α3K and the N-terminus of the GlyR α3 TM4 domain (Figure 2A). However, in contrast to the constructs used in Figure 1, the fluorescent reporter protein was inserted upstream of the C-to-U RNA editing sensor GlyR TM3-4 domain to indicate Apobec-1-dependent RNA editing through translocation of the fluorescence into the nucleus. Editing of the CAA codon to the STOP codon UAA should thus result in nuclear targeting of the reporter fluorescence protein (here EGFP upstream of the sensor domain). In order to test the efficacy of the editing sensor, we used the recently established faithful multicistronic co-expression technology (Tang et al., 2009). Editing sensor was either expressed alone or in combination with human Apobec-1 complementation factor (ACF) and Apobec-1 80I or 80M variants, using the *Thosea asigna* virus 2A self-processing peptides (Tang et al., 2009; Figure 2B).

**Figure 3:**
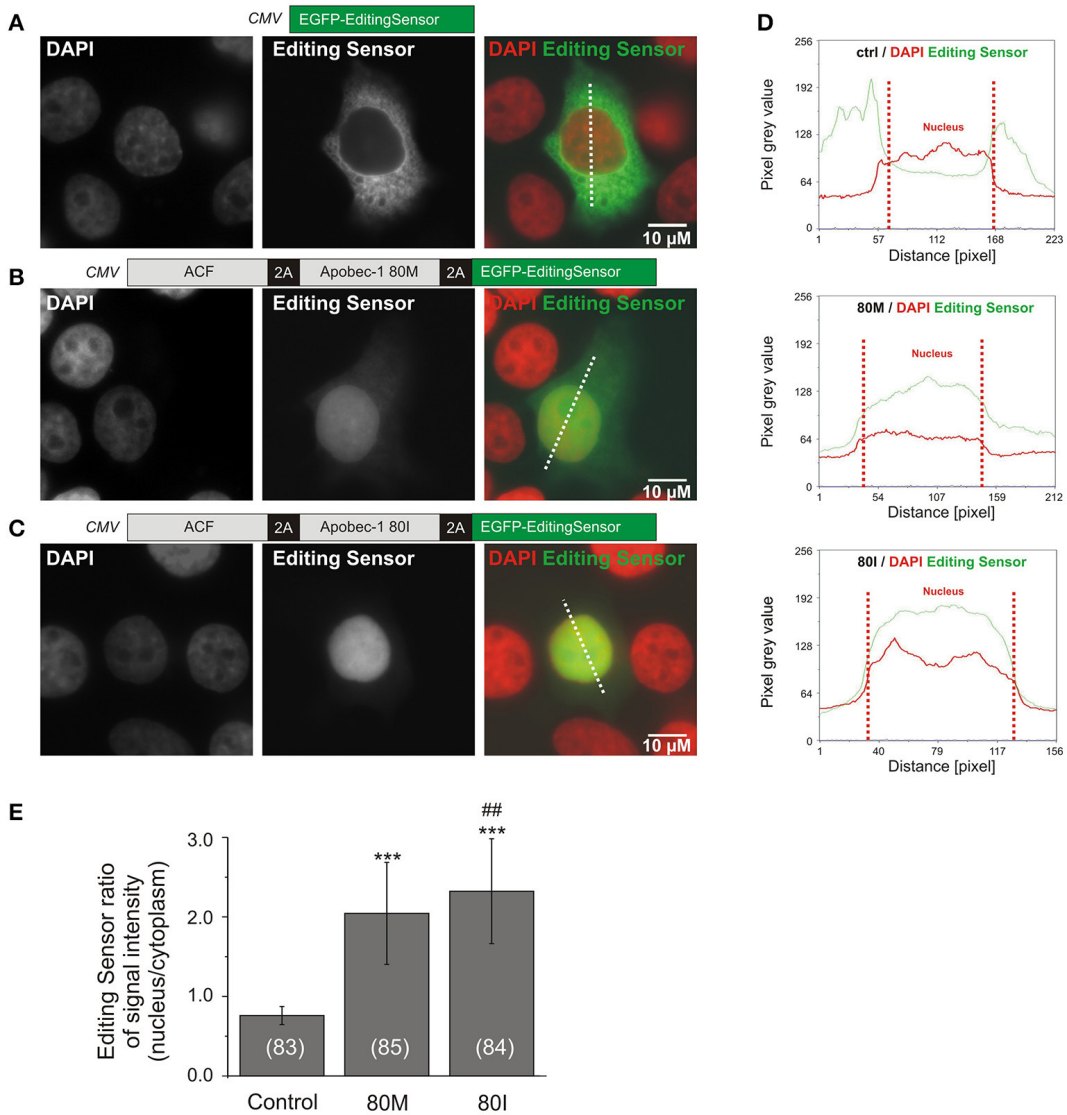
The C-to-U RNA editing deficient HEPG2 liver carcinoma cell line was used to evaluate the functionality of the RNA editing sensor. **(A–C)** Representative images of HEPG2 cells transfected with the constructs shown in Figure 2B. Images show fluorescence corresponding to DAPI (left panels), sensor signals (middle panels), and merged signals (right panels) without (**A**, control, editing sensor alone) or with Apobec-1 80M (**B**, 80M) or Apobec-1 80I (**C**, 80I) co-expression. Dotted lines show examples of the placement of line-scans shown in **(D)**. **(D)** Line scans of pixel intensities of the editing sensor (green) and DAPI (red) through cytosol and nucleus in the three different conditions are shown. Dotted red vertical lines delineate the nuclear region in each case (“Nucleus”). **(E)** Quantification of the fluorescence ratios between nucleus and cytosol in line-scanned specimen. All data are given as mean ± SD. Asterisks mark significant differences between editing sensor alone and with 80M or 80I. The “##” symbol indicates a significant difference (*P* = 0.006) between 80M and 80I while ^***^ mark significant differences between control and 80M or 80I (*P* < 0.001), as assessed using Mann-Whitney test. The number of analyzed cells is indicated in brackets.

We investigated the functionality of these constructs using the C-to-U RNA editing-deficient liver carcinoma cell line HEPG2 (Chen et al., 2010). As Apobec-1 was previously associated with C-to-U RNA editing of apolipoprotein B and GlyR (Backus and Smith, 1992; Meier et al., 2005), we assessed the effect of Apobec-1 80M and 80I on the subcellular localization of the editing sensor. Figures 3A–C show representative images of HepG2 cells expressing EGFP-editing sensor (A, control) or ACF-Apobec-1 80M/I-editing sensor constructs shown in Figures 2B, 3B,C. Quantification using line scans of fluorescence intensities in cytosol and nucleus of the transfected cells (Figure 3D) corroborated the marked first impression of prominent nuclear targeting of the sensor upon 2A-peptide-dependent co-expression of 80M or 80I Apobec-1 variants [mean fluorescence ratios between nucleus and cytosol; editing sensor alone: 0.761 ± 0.114 (mean ± SD), editing sensor with Apobec-1 80M or 80I: 2.031 ± 0.646 (mean ± SD) and 2.324 ± 0.659 (mean ± SD), respectively; Figure 3E].

**Figure 4:**
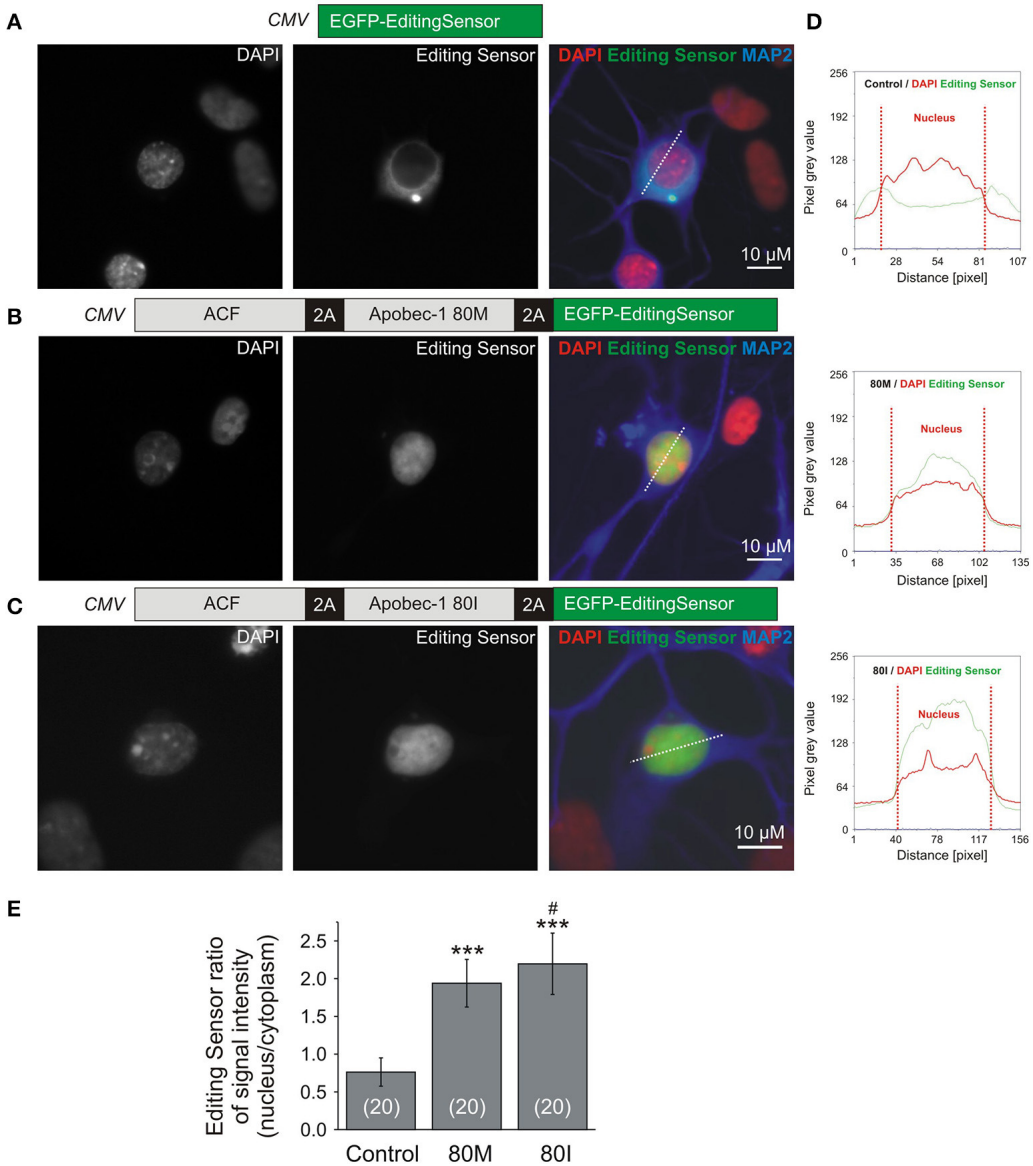
Primary rat hippocampal (RHC) neurons were used to evaluate the functionality of the RNA editing sensor. **(A–C)** Representative images of RHC neurons transfected with the constructs shown in Figure 2B. Images show fluorescence corresponding to DAPI (left panels), sensor signals (middle panels), and merged signals including MAP2-stain shown in blue (right panels) without (**A**, control, editing sensor alone) or with Apobec-1 80M (**B**, 80M) or Apobec-1 80I (**C**, 80I) co-expression. Dotted lines show examples of the placement of line-scans quantified in **(E)**. **(D)** Examples of line scans of pixel intensities of the editing sensor (green) and DAPI (red) through cytosol and nucleus in the three different conditions. The dotted red vertical lines delineate the nuclear region (“Nucleus”). **(E)** Quantification of the fluorescence ratios between nucleus and cytosol in line-scanned specimen. All data are given as mean ± SD. Asterisks mark significant differences between editing sensor alone and with 80M or 80I. The “#” symbol indicates a significant difference (*P* = 0.034) between 80M and 80I while ^***^ mark significant differences between control and 80M or 80I (*P* < 0.001), as assessed using one-way Anova followed by *post-hoc* Tukey test. The number of analyzed cells is indicated in brackets.

To pursue these promising results, we next characterized the performance of the editing sensor in rat primary hippocampal neurons (Figure 4). Figures 4A–C shows representative images of primary neurons expressing EGFP-editing sensor (Figure 4A, control) or ACF-Apobec-1 80M/I-editing sensor constructs shown in Figures 2B, 4B,C. As in HEPG2 cells, quantification using line scans of fluorescence intensities in cytosol and nucleus of the transfected neurons (Figure 4D) revealed that nuclear targeting of the editing sensor was increased upon 2A-peptide-dependent co-expression of 80M or 80I Apobec-1 variants [mean fluorescence ratios between nucleus and cytosol; editing sensor alone: 0.763 ± 0.187 (mean ± SD), editing sensor with Apobec-1 80M or 80I: 1.940 ± 0.316 and 2.197 ± 0.407 (mean ± SD), respectively; Figure 4E].

**Figure 5:**
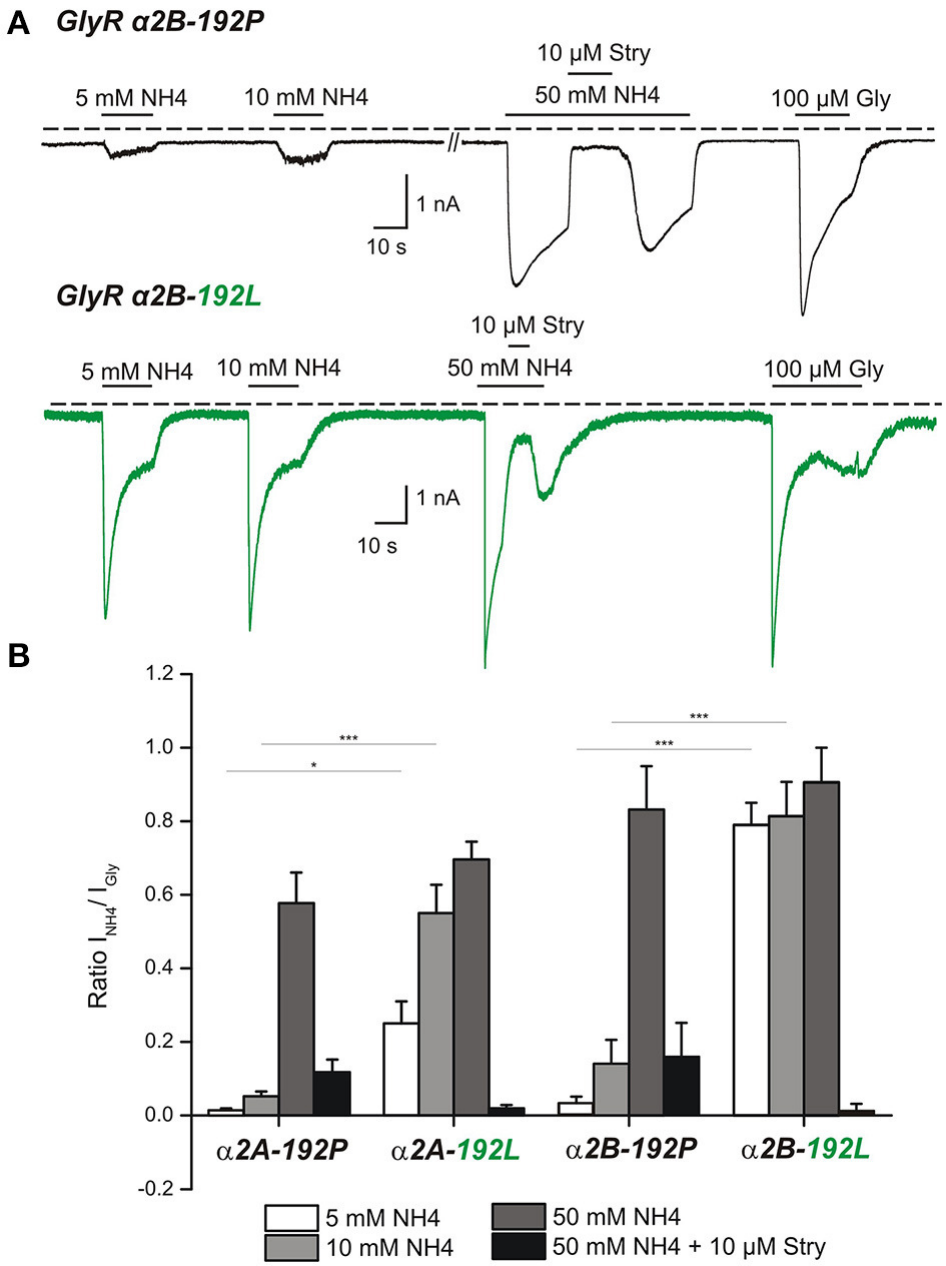
Whole cell patch clamp analysis of transfected HEK293T cells reveals the potential of NH_4_ to activate RNA-edited, not the non-edited, GlyR α2 at physiological concentrations. **(A)** HEK293T cells were transfected with non-edited GlyR α2 (192P, upper panel) or C-to-U RNA-edited GlyR α2 (192L, lower panel). Traces of electrophysiological recordings show NH_4_ dose-dependent effects on GlyR currents. Note that 5 and 10 mM NH_4_ resulted in significantly stronger activation of RNA-edited GlyR α2 compared to non-edited GlyR α2. At a concentration of 50 mM NH_4_ both types of receptors were comparably activated. Currents could be blocked with 10 μM strychnine (Stry). For normalization, current responses to 100 μM glycine were acquired in the same cells. **(B)** Quantification of NH_4_-elicited currents relative to currents elicited with 100 μM glycine. Asterisks mark significant differences (^*^P <0.05, ^***^P < 0.001), as assessed using one-way Anova followed by *post-hoc* Tukey test. For information about the number of analyzed cells see Table 2.

Our pharmacological screens (Schneidereit et al., 2017) suggested NH_4_ as agonist of RNA-edited GlyRs, which may allow discrimination of non-edited and edited GlyRs. To address this possibility in detail, we used transfected HEK293T cells and performed whole cell patch clamp analysis (Figure 5). We focused here on the GlyR α2 subunit because primary hippocampal neurons at DIV8-13 predominantly express GlyR α2 (Meier and Grantyn, 2004; Raltschev et al., 2016). However, as both splice variants of GlyR α2 (Figures 5A,B) and the GlyR α3L subunit are expressed in the iTLE hippocampus (Eichler et al., 2008), we included both GlyR α2 splice variants (Figure 5B) and GlyR α3L (Supplementary Figure 1) into our analyses. Thus, we co-expressed EGFP and GlyR α2A-192P/L, α2B-192P/L, or α3L-185P/L in HEK293T cells and recorded the currents in response to 5–50 mM NH_4_ and 100 μM glycine (Figure 5, Supplementary Figure 1). Quantification of the current ratios revealed that 10 mM NH_4_ is the most suitable concentration for effective discrimination between non-edited and edited α2- and α3-GlyRs (Figure 5B, Supplementary Figure 1). Furthermore, strychnine blocked NH_4_-elicited currents, indicating that NH_4_ indeed activated GlyRs (for values see Table 2, Supplementary Table 1).

**Table 2:** Whole cell patch clamp recording of transfected HEK293T cells.

	**5 mM (192P)**	**10 mM (192P)**	**50 mM (192P)**	**50 mM + 10 μM Stry (192P)**	**5 mM (192L)**	**10 mM (192L)**	**50 mM (192L)**	**50 mM + 10 μM Stry (192L)**
N	11	12	9	8	12	13	7	11
Mean	0.013	0.052	0.577	0.117	0.250	0.550	0.696	0.019
SD	0.020	0.045	0.241	0.098	0.208	0.278	0.126	0.031
SEM	0.006	0.013	0.080	0.035	0.060	0.077	0.048	0.009
N	9	9	9	9	9	9	9	8
Mean	0.030	0.139	0.832	0.161	0.789	0.814	0.901	0.014
SD	0.055	0.194	0.355	0.273	0.179	0.279	0.282	0.043
SEM	0.018	0.065	0.118	0.091	0.059	0.092	0.094	0.015

*Applied NH_4_ concentration is indicated in mM. The upper part of the table represents current response ratios to 100 μM glycine and the indicated NH_4_ concentrations in cells expressing the GlyR α2A splice variant, while the lower part shows characteristics of GlyR α2B-expressing cells. “192P” denotes conditions with non-edited GlyR expression, while “192L” indicates cells expressing the RNA-edited GlyR. N, number of cells; SD, standard deviation; SEM, standard error of the mean; Stry, strychnine*.

**Figure 6:**
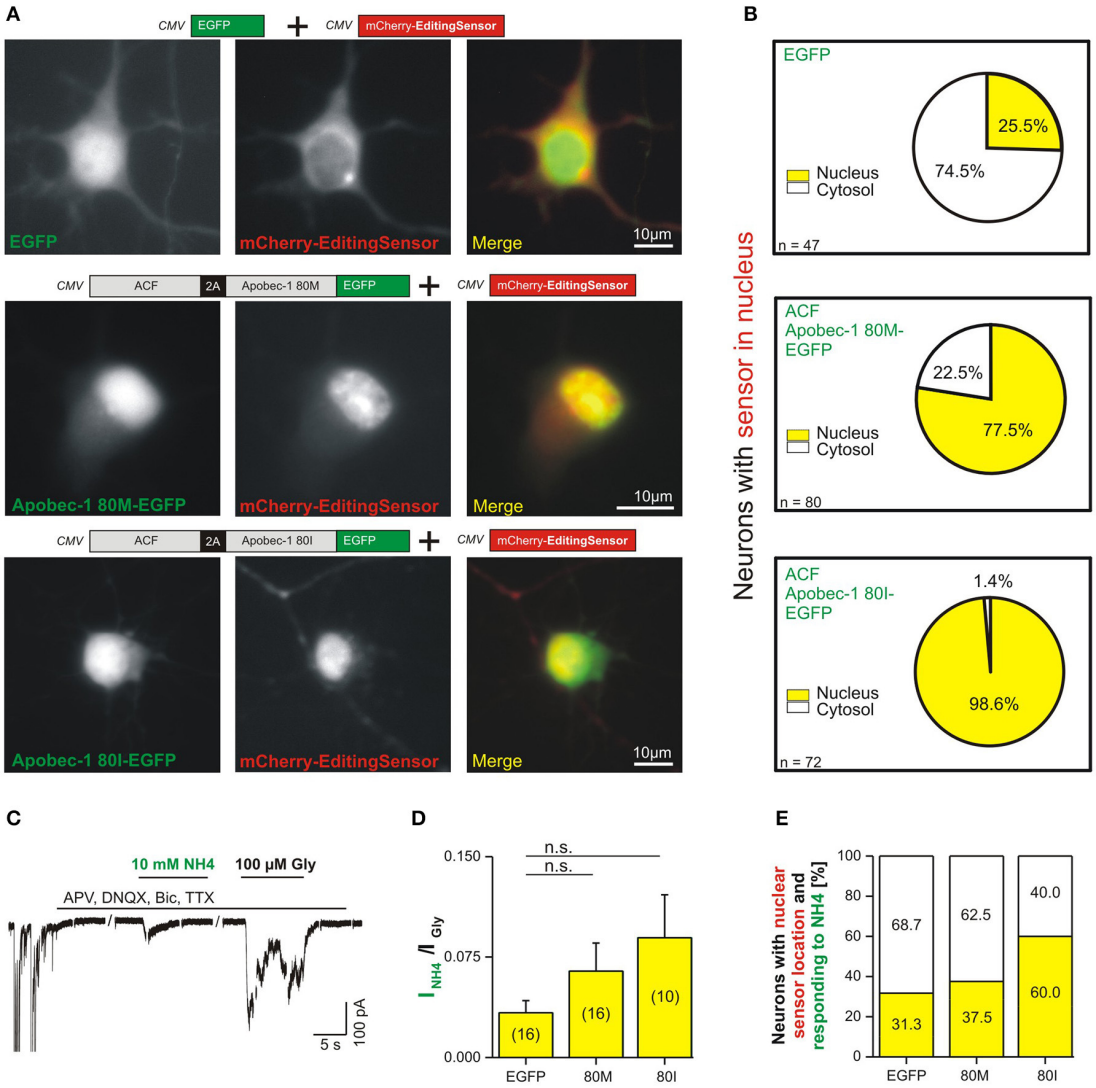
Application of 10 mM NH_4_ reveals endogenous expression of RNA-edited GlyR protein in primary hippocampal neurons. **(A)** We co-transfected here the mCherry editing sensor either without Apobec-1 (EGFP, control) or with EGFP-tagged ACF-Apobec-1 80M or 80I to check the nuclear localization of the ectopically expressed human Apobec-1 protein in RHC neurons. Representative images document preferential cytosolic localization of the editing sensor in neurons co-transfected with EGFP (control, upper panel). In contrast, co-expression of Apobec-1 80I or 80M variants triggered nuclear localization of the editing sensor. **(B)** Quantification of the fraction of neurons with editing sensor in nucleus vs. cytosol in the three different conditions. **(C)** The example trace shows NH_4_ responsiveness of a neuron expressing Apobec-1 80I. Spontaneous neuronal activity was acquired for control purpose (left-hand) and blocked using TTX, APV and DNQX, and bicuculline (BIC) during acquisition of responses to 10 mM NH_4_. **(D)** Quantification of the ratio of peak amplitudes of 10 mM NH_4_- and 100 μM glycine-evoked currents in EGFP (control) or Apobec-1 80M or 80I overexpressing neurons with nuclear sensor localization, representing C-to-U RNA editing-competent neurons. Statistical analysis using Mann-Whitney test did not reveal significant differences (“n.s.”, *P* > 0.05); the number of investigated neurons is indicated in brackets. **(E)** Quantification of the fraction of neurons with nuclear editing sensor localization and NH_4_-responsiveness reveals an Apobec-1 80I-dependent increase of the expression of RNA-edited GlyR protein (31.3 vs. 60.0%). Note that a NH_4_-responsive neuron was determined if the amplitude ratio between 10 mM NH_4_- and 100 μM glycine-evoked currents was above 0.033 (EGFP control condition).

To investigate endogenous expression of RNA-edited GlyR protein, we transfected primary hippocampal neurons with the editing sensor, in which EGFP was substituted by mCherry (above, Figures 2–4), alone or in combination with ACF-Apobec-1 80M/I-EGFP variants (Figure 6A). As control, EGFP was co-transfected with the editing sensor. The co-transfection experimental approach was used here to detect subcellular localization of the Apobec-1 variants and the editing sensor simultaneously in live neurons. Figure 6A shows representative images of live neurons expressing mCherry editing sensor (middle panels) with either EGFP (control, upper row), Apobec-1 80M-EGFP (middle row), or Apobec-1 80I-EGFP (lower row). Analysis of the fraction of neurons with nuclear sensor localization revealed that 25.5% of EGFP-expressing neurons had a nuclear sensor localization (12 out of 47; Figure 6B, upper row). Upon coexpression of Apobec-1 80M, 77.5% (62 out of 80) showed a nuclear sensor localization (Figure 6B, middle row), whereas 98.6% (71 out of 72) of neurons showed nuclear sensor localization upon Apobec-1 80I expression (Figure 6B, lower row). We finally tested neurons with nuclear sensor localization for NH_4_ responsiveness (Figures 6C–E). In the EGFP control group, the mean ratio between current amplitudes elicited by 10 mM NH_4_ and 100 μM glycine was 0.033 ± 0.009 (*n* = 16; Figure 6D). Both, Apobec-1 80M and 80I variants increased this ratio to 0.064 ± 0.021 (*n* = 16) and 0.089 ± 0.032 (*n* = 10), respectively (Figure 6D). The fraction of NH_4_-responsiveness was then calculated according to amplitude ratios between NH_4_- and glycine-dependent responses that were above 0.033, which corresponded to the mean ratio we measured in the EGFP control group (Figure 6E). Although slightly increased in both 80M and 80I conditions, the ratios between current amplitudes elicited by 10 mM NH_4_ and 100 μM glycine in NH_4_-responsive neurons was not significantly different (control: 0.076 ± 0.016, *n* = 5; 80M: 0.151 ± 0.033, *n* = 6; 80I: 0.140 ± 0.042, *n* = 6). However, this revealed that Apobec-1 80I almost doubled the fraction of NH_4_-responsive neurons (60%, 6 out of 10), compared to control neurons (EGFP, 31.25%, 5 out of 16 neurons) or neurons with Apobec-1 80M expression (37.5%, 6 out of 16; Figure 6E). Thus, human Apobec-1 80I is more efficient than the 80M variant in generating RNA-edited GlyR protein in primary rat hippocampal neurons.

**Figure 7:**
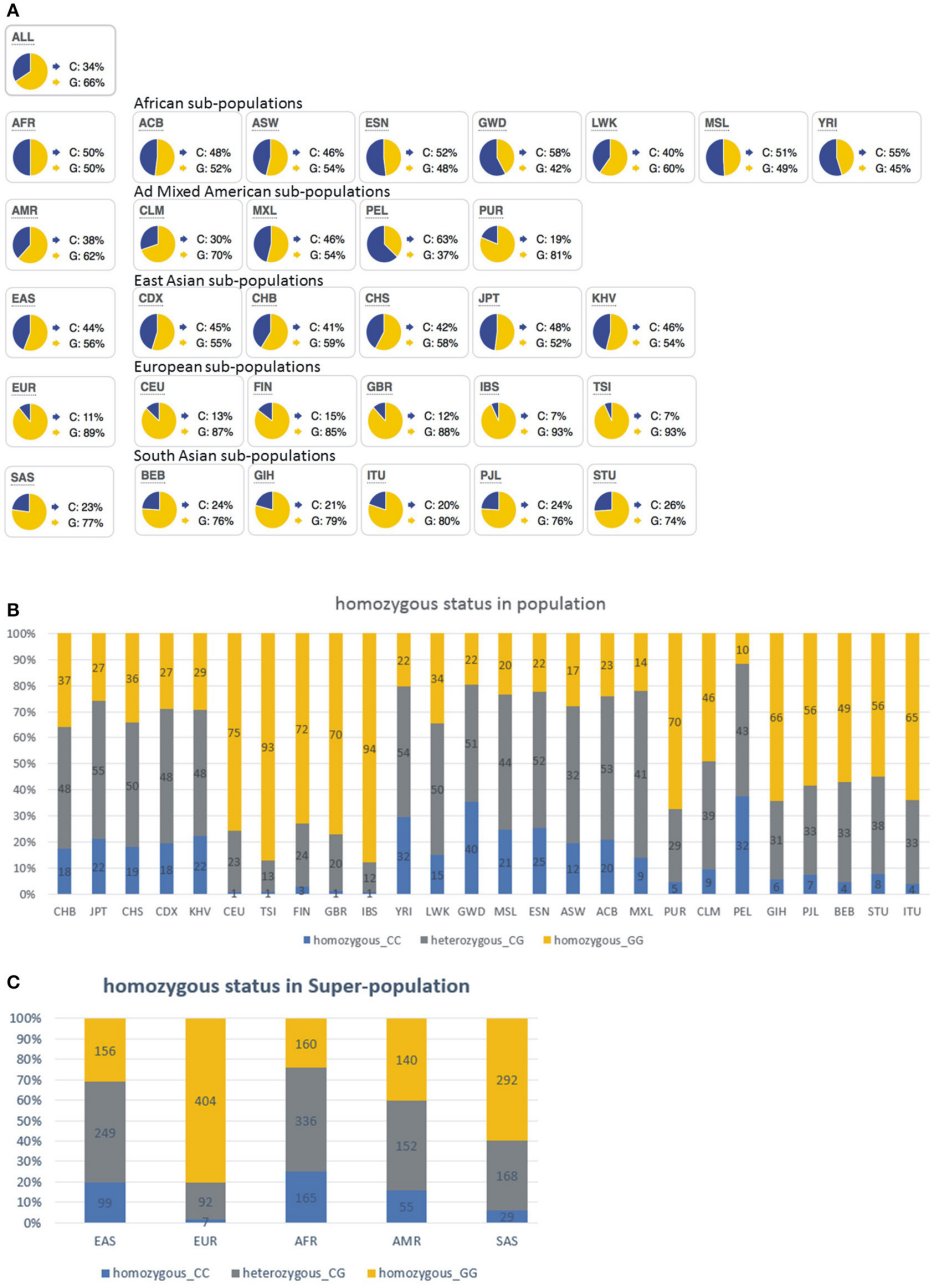
Bioinformatic analysis of 1000 Genome Project Phase 3 allele frequencies coding for human Apobec-1 80M and 80I variants reveal world-wide differences. **(A)** Note that the C allele codes for 80M (shown in blue) while the G allele codes for 80I (shown in yellow). Details about frequencies are shown in Supplementary Table 2. SNP: rs2302515. Ref allele: C, ancestral allele: G. This analysis indicates that the European population predominantly encodes the 80I-Apobec-1 variant, compared to, for example, only 50% of the African population. **(B)** Representation of homo- and heterozygosity of APOBEC1 80M- and 80I- coding alleles. Bar graph shows the frequency of the alleles in populations, with the number showing the sequenced alleles in phase 3 of the 1,000 Genomes Project. Homozygous 80M-coding (CC, blue), heterozygous 80M-/80I-coding (CG, gray), and Homozygous 80I-coding (GG, yellow). **(C)** Representation of homo- and heterozygosity of APOBEC1 80M- and 80I- coding alleles in super-population. Bar graph shows the frequency of the alleles in populations, with the number showing the sequenced alleles in phase 3 of the 1000 Genomes Project. Homozygous 80M-coding (CC, blue), heterozygous 80M-/80I-coding (CG, gray), and homozygous 80I-coding (GG, yellow).

Two different genomic human *APOBEC1* variants are annotated (NCBI Reference Sequences NM_001644.4 and AAA64230.1 coding for 80M and 80I, respectively). Through a bioinformatic analysis of 1,000 Genome Project Phase 3 allele frequencies we investigated the world-wide distribution of genotypes corresponding to *APOBEC1* 80M- and 80I-coding alleles (Figure 7, Supplementary Table 2; note that the human *APOBEC1* reference gene sequence is annotated in antisense direction of the ORF-coding sequence). This analysis revealed that the 80I-coding allele is most frequent world-wide (Figure 7A, “ALL,” G: 66%). The European population leads with 89% of individuals harboring the 80I-coding allele (Figure 7A, “EUR”), followed by the South Asian population (Figure 7A, “SAS,” G: 77%), the American population (Figure 7A, “AMR,” G: 62%), East Asian and African population (Figure 7A, “EAS,” “AFR,” G: 56%, and 50%, respectively). Figures 7B,C reveal the distribution of homo- and heterozygous allele carriers. The broader European population (EUR) featured 496 out of 503 reads (98.6%) which detected at least one allele coding for 80I; while the frequency drops to 75.0% in the African population (AFR); see Supplementary Table 2 for details.

**Figure 8:**
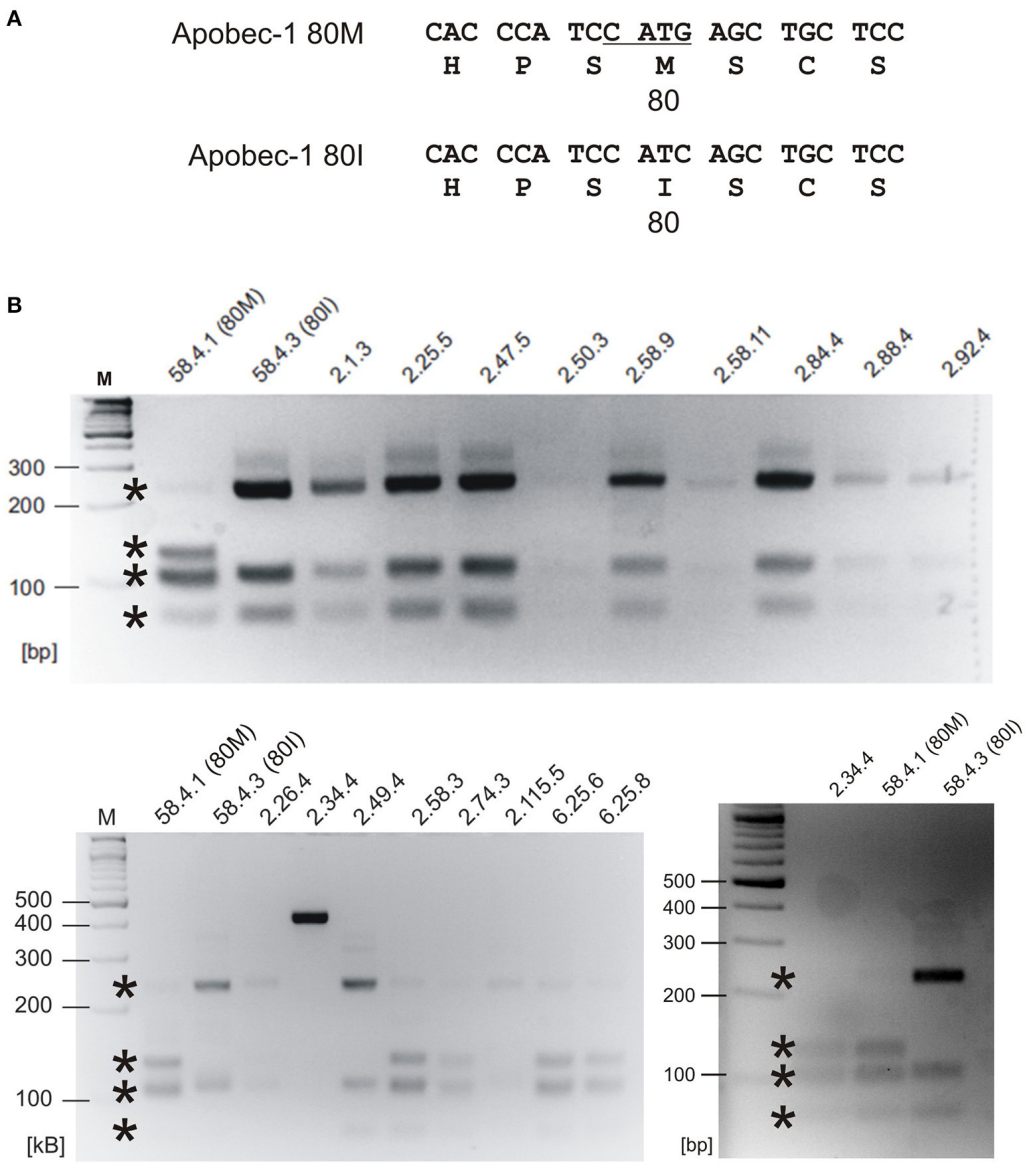
Retrospective *APOBEC1* mRNA expression profiling of iTLE patients. **(A)** Sequences of human Apobec-1 80M and 80I including NlaIII restriction site in the 80M variant (underlined) are shown. Amino acid sequence is shown in single letter code. **(B)** NlaIII digest results in bands of the following sizes (in bps, and indicated by asterisks). 80M: 73, 102, 107, 129; 80I: 73, 107, 231. The agarose gels show expected DNA bands resulting from restriction digest with NlaIII. For control purpose, 80M- and 80I-coding cDNA clones (58.4.1 and 58.4.3, respectively) were amplified and processed in parallel. Upper and lower panels: representative analysis of Apobec-1 80I and 80M expression, respectively. Note that assessment of patient ID 2.34.4 was repeated because restriction digest failed in the first run. *M* = gene ruler DNA size marker. bp = base pairs.

Given the world-wide heterogeneity of 80I- and 80M-coding *APOBEC1* allele frequencies and regarding the preference of the Apobec-1 80I variant to generate RNA-edited GlyR protein, we wondered whether iTLE patients analyzed in 2008 with regard to GlyR RNA editing (Eichler et al., 2008) expressed the 80I- or 80M-coding allele. For this purpose, we developed an assay for detecting Apobec-1-coding mRNAs. The 80M-coding sequence gives rise to an NlaIII restriction site CATG and thus allows discrimination of 80M- and 80I-coding *APOBEC1* gene transcripts according to different restriction fragment lengths (Figure 8A). As control and restriction fragment lengths reference, cDNA clones encoding Apobec-1 80M or 80I were processed in parallel, and GAPDH was amplified to ensure integrity of cDNA probes. We analyzed the 23 iTLE samples published in 2008 (see Table 1 in Eichler et al., 2008 for details on iTLE patients). Representative examples of the results of the PCR-RFLP experiments are shown in Figure 8B. Nineteen out of 23 samples showed Apobec-1 mRNA expression. In detail, 14 out of 19 expressed 80I-coding mRNA, and 5 out of 19 expressed 80M-coding mRNA. Thus, a fraction of 74% expressed the 80I-coding allele, which is lower than expected according to the data provided by our bioinformatics analysis (above, Figures 7B,C). With regard to anamnesis data of the iTLE patients analyzed in 2008, we found that the majority of patients (13 of 14) with Apobec-1 80I expression (93%) suffered from simple and/or complex partial seizure activity. In contrast, 2 out of 5 iTLE patients with expression of the 80M allele (40%) experienced secondarily generalized tonic clonic seizures, with 4 out of the 5 iTLE patients with 80M expression belonging to the W2-4 group (Eichler et al., 2008).

## Discussion

Changes in neural network homeostasis due to maladaptive changes in presynaptic plasticity and resulting persistent neuronal gain or loss of function can play a critical role in neuropsychiatric comorbidities of iTLE. In more detail, we showed that presynaptic expression of gain-of-function C-to-U RNA-edited GlyR leads to neuronal gain of function and, depending on the affected neuron type, elicits either cognitive dysfunction or persistence of contextual fear memory (Meier et al., 2014, 2016; Winkelmann et al., 2014; Çaliskan et al., 2016). Recent advances in sequencing technologies provide insights into global changes in RNA editing, but they are not able to draw conclusions on the cell/neuron type-specific changes involved in maladaptive plasticity in disease as they use bulk material. Thus, innovative technologies are required to assess changes in posttranscriptional RNA processing at the single cell level. Here, we present novel molecular and chemical tools for the investigation of changes in C-to-U RNA editing at the single cell level. Furthermore, our results highlight a possible link between Apobec-1 80I expression and simple or complex partial seizure activity.

Our molecular sensor tool indicates C-to-U RNA editing by nuclear translocation of a fluorescence protein, which provides a major advance compared to a recently published alternative tool that involves kinetics of EGFP protein turnover (Severi and Conticello, 2015). Our tool does not involve protein stability but fast kinetics of nuclear import at a time scale of seconds (Förstera et al., 2014). Thus, our sensor tool will be applicable to assess acute changes in Apobec-1 enzyme activity. Indeed, our new tool reliably indicated C-to-U RNA editing: Equimolar co-expression of the editing sensor and 2A-peptide-dependent Apobec-1 80M and 80I variants demonstrated efficient nuclear translocation of the sensor protein in the C-to-U RNA editing-deficient cell line HEPG2 (Figure 3). Moreover, we showed that our editing sensor works equally well in transfected rat primary hippocampal neurons (Figure 4). Our live cell imaging experiments furthermore revealed that spontaneous C-to-U RNA editing by the rat Apobec-1 80T variant (in the absence of co-expressed human Apobec-1) occurred in about 25% of sensor-expressing primary hippocampal neurons. In fact, our experiments revealed that expression of human Apobec-1 80I and the auxiliary protein ACF rendered almost all 80I-expressing neurons editing competent (Figure 6), and they showed that the 80I variant was significantly more effective in translocating the sensor to the neuronal nucleus compared to the 80M variant (Figure 4E).

The C-to-U RNA editing sensor tool presented here reliably indicated RNA editing in HEPG2 cells and primary neurons, but it did not allow to draw conclusions on GlyR C-to-U RNA editing. For this purpose, a combined approach involving NH_4_ action on RNA-edited GlyRs and the sensor tool was required. Indeed, we showed that application of 10 mM NH_4_ elicits strychnine-sensitive RNA-edited GlyR-dependent currents in transfected HEK293T cells. However, the fractions of C-to-U RNA editing-competent and NH_4_-responsive neurons in the context of rat endogenous neuronal Apobec-1 80T expression or human Apobec-1 80M overexpression were rather low (31.3 vs. 37.5%). Overexpression of the human Apobec-1 80I variant increased this fraction to about 60% of neurons with nuclear sensor localization. This result reveals a preference of Apobec-1 80I for GlyR α2 C-to-U RNA editing and indicates that neuronal GlyR α2 C-to-U RNA editing is regulated, depending on the amino acid at position 80 of the polypeptide chain of Apobec-1. The ratio between current amplitudes generated by 10 mM NH_4_ and 100 μM glycine in transfected HEK293T cells with RNA-edited GlyR α2-192L was about 10 times higher compared to neurons that overexpressed Apobec-1 80M or 80I in primary rat hippocampal neurons (Figure 5 vs. Figure 6). This supports neuronal endogenous regulation of GlyR C-to-U RNA editing and furthermore indicates that about 10% of endogenous rat neuronal GlyR α2 were edited in NH4-responsive and C-to-U editing-competent neurons. This fraction corresponds well with the fraction of RNA-edited GlyR α2 in iTLE patients with a severe course of disease (Eichler et al., 2008). However, all the other C-to-U editing enzymes expressed in primary rat hippocampal neurons may also contribute to neuronal regulation of GlyR α2 RNA editing, even irrespectively of ACF (Supplementary Figure 2). In agreement with a recent study (Snyder et al., 2017), co-expression of ACF with Apobec-1 80M or 80I did not affect the preference of Apobec-1 80I over 80M for GlyR α2 C-to-U RNA editing.

The preference of Apobec-1 80I over 80M for expression of GlyR α2 C-to-U RNA-edited protein in neurons led us to develop a new approach for the retrospective study of mRNA expression of Apobec-1 80M and 80I in iTLE samples (Eichler et al., 2008). Based on the fact that the 80M-coding sequence generates an NlaIII restriction site, we investigated mRNA preparations of previously characterized iTLE patients (Eichler et al., 2008). We chose investigation of mRNA derived from these iTLE patients because this approach unambiguously provides information about Apobec-1 mRNA expression even if a heterozygous genetic *APOBEC1* background exists. In contrast to the rather low overall representation of the *APOBEC1* 80M-coding allele in our analysis of allele frequency distributions in Europe (11%, Figure 7A), assessment of heterozygosity in this population revealed a higher fraction (20%) of persons with at least one allele that codes for the 80M Apobec-1 variant (Figure 7C, see Supplementary Table 2 for details). Departing from this allele-distribution in our sample of the general European population, 26% of the iTLE patients analyzed in 2008 expressed the 80M allele, and 4 out of 5 iTLE patients with 80M expression belong to the W2-4 group of iTLE patients with increased expression of RNA-edited GlyR (Eichler et al., 2008). Although we can't retrospectively provide information regarding the ethnic origin of these patients, this result suggests an overrepresentation of iTLE patients with 80M expression compared to our bioinformatics analysis (26 vs. 20% in Europe), and the mismatch between increased GlyR RNA editing in the W2-4 group of iTLE patients with Apobec-1 80M expression suggests that other C-to-U RNA editing enzymes have contributed to GlyR RNA editing (including the GlyR α3 subunit) in these patients. Furthermore, pronounced hippocampal cell loss and the type of seizures in the W2-4 group of iTLE patients with Apobec-1 80M expression suggests that Apobec-1 80M targeted gene products are involved in neurodegeneration and secondary generalization of seizure activity (SGTCS). In fact, the success rate of our patch clamp analysis of neurons with 80M expression was rather low (38.1%), compared to 60.5% success rate in patch clamp experiments involving the 80I variant, and 50% of W2-4 classified iTLE patients with Apobec-1 80M expression experienced SGTCS. In contrast, the vast majority (93%) of the iTLE patients with Apobec-1 80I mRNA expression had simple or complex partial seizures. This fits well with the observed phenotypes of animals with increased expression of RNA-edited GlyR which exhibit cognitive dysfunction and impairment of spatial and contextual fear memory (Winkelmann et al., 2014; Çaliskan et al., 2016). Considering the rather low fraction of RNA-edited GlyRs in patients with iTLE and assuming a uniform receptor distribution in all neuronal compartments supports the proposed pathogenic presynaptic role of these receptors in response to low extracellular glycine concentrations, due to the low capacitance of the presynaptic compartment and that irrespectively of whether RNA-edited GlyR α2 or α3 is expressed (Meier et al., 2014, 2016), which is in agreement with a previous study (Chen et al., 2014). However, it is possible that only a few neurons or certain neuron types generate larger amounts of RNA-edited GlyRs in iTLE and contribute in a neuron type-specific way to different symptoms (Winkelmann et al., 2014; Çaliskan et al., 2016). We cannot rule out this possibility yet because bulk material was used for investigation of GlyR α2 or α3 RNA editing in iTLE (Eichler et al., 2008).

In conclusion, our study revealed human genetic *APOBEC1* 80M/I dimorphism as a new diagnostic marker in iTLE. The 80I variant can contribute to simple or complex seizure activities and increases protein expression of RNA-edited GlyR, which would preferentially affect the presynaptic compartment and hence trigger maladaptive and persistent dysregulation of neuron gain control, resulting in simple or complex partial seizures. In contrast, the 80M Apobec-1 variant is suggested to promote neurodegeneration and secondary generalization of seizure activity. Moreover, the results indicate that a complex interplay of different C-to-U RNA editing enzymes regulates GlyR RNA editing in iTLE, which remains to be characterized at the single cell level for example by applying the technologies established here to resected hippocampi from patients with iTLE. Finally, this study provides the basis for a new mapping of epidemiology and semiology of iTLE with regard to *APOBEC1* 80M/I alleles, which can open avenues for further characterization of the functional roles of Apobec-1 80M and 80I variants in healthy and diseased brains.

## Ethics statement

This study was carried out in accordance with the recommendations of guidelines of the Charité Ethics Commission (EA1/142/05) with written informed consent from all subjects. All subjects gave written informed consent in accordance with the Declaration of Helsinki. The protocol was approved by the Charité Ethics Commission.

## Author contributions

SK, BF, AW, PK, XY, MS, EW, and FH performed experiments. SK, FH, and JM wrote the manuscript. JM designed the study.

### Conflict of interest statement

The authors declare that the research was conducted in the absence of any commercial or financial relationships that could be construed as a potential conflict of interest.
