# Corrigendum: A Novel RNA Editing Sensor Tool and a Specific Agonist Determine Neuronal Protein Expression of RNA-Edited Glycine Receptors and Identify a Genomic APOBEC1 Dimorphism as a New Genetic Risk Factor of Epilepsy

**DOI:** 10.3389/fnmol.2019.00103

**Published:** 2019-04-24

**Authors:** Svenja Kankowski, Benjamin Förstera, Aline Winkelmann, Pina Knauff, Erich E. Wanker, Xintian A. You, Marcus Semtner, Florian Hetsch, Jochen C. Meier

**Affiliations:** ^1^Division Cell Physiology, Zoological Institute, Technische Universität Braunschweig, Braunschweig, Germany; ^2^Institute for Stroke and Dementia Research, Klinikum der Universität München, Ludwig Maximilians University of Munich, Munich, Germany; ^3^Neuroproteomics, Max Delbrueck Center for Molecular Medicine, Berlin, Germany; ^4^Institute of Cell Biology and Neurobiology, Charité Universitätsmedizin Berlin, Berlin, Germany; ^5^Bioinformatics in Medicine, Zuse Institute Berlin, Berlin, Germany; ^6^Cellular Neurosciences, Max Delbrueck Center for Molecular Medicine, Berlin, Germany

**Keywords:** glycine receptors, epilepsy, temporal lobe, RNA editing, hippocampus, ligands

In the original article, there was an error. The incorrect oligonucleotide sequences were provided.

A correction has been made to the **Methods**, subsection **PCR-RFLP Analysis of Human TLE Samples**:

“Resected hippocampal tissue of human iTLE patients (Eichler et al., 2008) was analyzed with regard to *APOBEC1* gene dimorphism coding for 80M or 80I Apobec-1 protein variants. For this purpose, we developed a new PCR-based RFLP approach. Total RNA was isolated and reverse transcribed into cDNA as described earlier (Raltschev et al., 2016). Pre-amplification of Apobec-1 was performed using oligonucleotides 5′-CTTCAACCGGTGACCCCACTC-3′ and 5′-TGCGTACAACATCATCCACAGAGG-3′. Then, 3.5 μl of the pre-PCR were investigated in another PCR using oligonucleotides 5′-GAGTTTGACGTCTTCTATGACCC-3′ and 5′-GTTGACAAAATTCCTCCAGCAG-3′ to amplify a region spanning the 80M/I-coding position. This nested PCR amplification step yielded sufficient amount of DNA that was purified with Monarch® DNA Gel Extraction Kit (catalog no. L1020L, New England Biolabs GmbH) and digested using NlaIII restriction enzyme. NlaIII cuts at the 80M-coding position (CATG), and restriction fragments were separated using electrophoresis with 5% agarose gels to identify the genotype of the iTLE patients. For control purpose, Apobec-1 80I- or 80M-coding vectors for transfection were processed in parallel. Ethidium bromide was used to stain DNA bands.”

The authors apologize for this error and state that this does not change the scientific conclusions of the article in any way. The original article has been updated.
