# Supplementary material for: A Novel RNA Editing Sensor Tool and a Specific Agonist Determine Neuronal Protein Expression of RNA-Edited Glycine Receptors and Identify a Genomic APOBEC1 Dimorphism as a New Genetic Risk Factor of Epilepsy
Source: Front Mol Neurosci. 2018 Jan 11;10:439. doi: 10.3389/fnmol.2017.00439 (PMC5768626; doi:10.3389/fnmol.2017.00439)
Supplement: Supplementary file 1 [file Presentation1.pdf]

Suppl. Figure 1

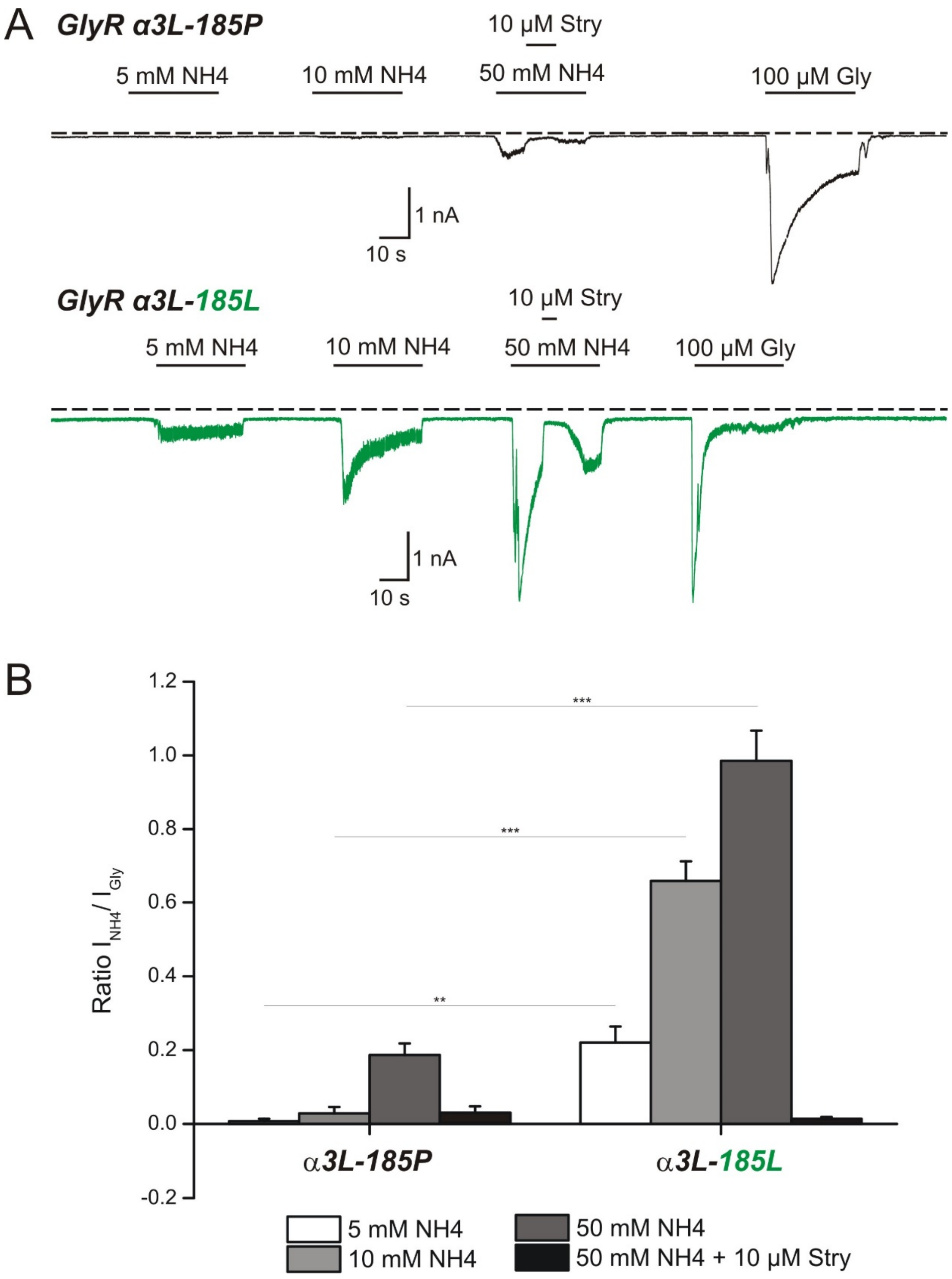

Suppl. Figure 1: Whole cell patch clamp analysis of transfected HEK293 reveal the potential of NH_4 to selectively activate RNA-edited GlyR α3L. (**A**) HEK293 cells were transfected with non-edited GlyR α3L (185P, upper panel) or C-to-U RNA-edited GlyR α3L (185L, lower panel). Traces of electrophysiological recordings show NH_4 dose-dependent effects on GlyR currents. Note that 10 mM NH_4 does not activate non-edited GlyR α3L, whereas it activates RNA-edited GlyR α3L. At a concentration of 50 mM NH_4 both types of receptors are activated albeit to much lesser extent for GlyR α3L-185P. Currents can be blocked with 10 μM strychnine (Stry). For normalization, current

responses to 100 µM glycine were acquired in the same cells. (**B**) Quantification of NH_4-elicited currents relative to currents elicited with 100 µM glycine. Asterisks mark significant differences (**: *P* < 0.01; ***: *P* < 0.001), as assessed using one-way Anova followed by post-hoc Tukey test. For values and number of investigated cells see Suppl. Table 1.

Suppl. Figure 2

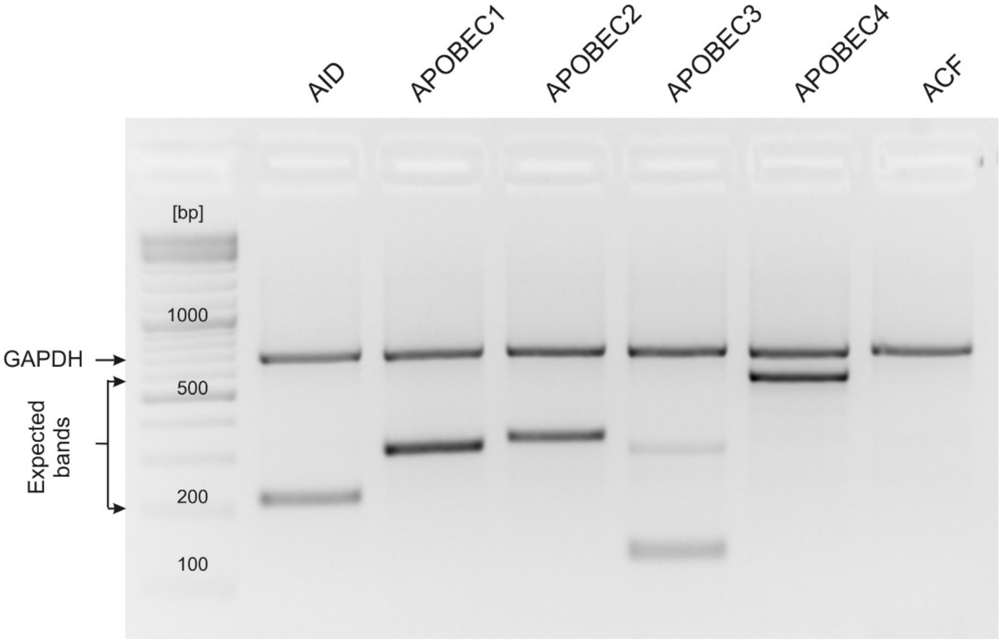

Suppl. Figure 2: The agarose gel shows PCR amplification products corresponding to GAPDH, activation-induced cytidine deaminase (AID), Apobec-1 to Apobec-4, and ACF. Size of bands of the DNA marker are indicated in base pairs (bp).

Suppl. Table 1:

Whole cell patch clamp recording of transfected HEK293 cells expressing GlyR α3L. The applied NH_4 concentration is indicated in mM. The table represents maximal current response ratios to 100 µM glycine and the indicated NH_4 concentrations in cells expressing the GlyR α3L splice variant. “185P” denotes conditions with non-edited GlyR expression, while “185L” indicates cells expressing the RNA-edited GlyR. N = number of cells, SD = standard deviation, SEM = standard error of the mean, Stry = strychnine.

**GlyR α3L**	**5 mM (185P)**	**10 mM (185P)**	**50 mM (185P)**	**50 mM + 10 µM Stry (185P)**	**5 mM (185L)**	**10 mM (185L)**	**50 mM (185L)**	**50 mM + 10 µM Stry (185L)**
**N**	10	10	10	10	11	11	9	11
**Mean**	0,008	0,029	0,187	0,033	0,221	0,658	0,985	0,014
**SD**	0,018	0,054	0,099	0,040	0,143	0,174	0,245	0,016
**SEM**	0,006	0,017	0,031	0,013	0,043	0,053	0,082	0,005

Suppl. Table 2: Frequencies corresponding to *APOBEC1* 80M- and 80I-coding alleles in the world.

THIS TABLE IS SUBMITTED AS SUPPLEMENTARY FILE named “315527_Meier_Data Sheet 1.XLSX”.
